# A Second-Order Disaster? Digital Technologies During the COVID-19 Pandemic

**DOI:** 10.1177/2056305120948168

**Published:** 2020-08-06

**Authors:** Mirca Madianou

**Affiliations:** Goldsmiths, University of London, UK

**Keywords:** COVID-19, social inequalities, digital welfare state, digital contact tracing, surveillance

## Abstract

One of the most striking features of the COVID-19 pandemic in the United Kingdom has been the disproportionate way in which it has affected Black, Asian, ethnic minority, and working class people. In this article, I argue that digital technologies and data practices in the response to COVID-19 amplify social inequalities, which are already accentuated by the pandemic, thus leading to a “second-order disaster”—a human-made disaster which further traps disadvantaged people into precarity. Inequalities are reproduced both in the everyday uses of technology for distance learning and remote work as well as in the public health response. Applications such as contact tracing apps raise concerns about “function creep”—the reuse of data for different purposes than the one for which they were originally collected—while they normalize surveillance which has been traditionally used on marginalized communities. The outsourcing of the digital public health response consolidates the arrival of the privatized digital welfare state, which increases risks of potential discrimination.

Pandemics are social and political as much as they are biological. COVID-19 is not just an infectious disease caused by the recently discovered coronavirus (SARS-Cov-2); the pandemic has affected all aspects of social life in most countries of the world. Because of its sudden arrival and severe infectiousness, COVID-19 has been likened to a disaster. But there are many reasons beyond its temporal urgency that have turned the pandemic into a disaster, at least in some countries. Disasters are not just unique, unexpected events: they have a longer temporality, involving the period predating the event and the subsequent recovery. Hurricane Katrina, which hit New Orleans in 2005, is a case in point. What turned the storm into a disaster were the underlying social and racial inequalities, the decaying public infrastructure, and the inadequate response which prioritized profit-making over the welfare of citizens (Adams, 2013). Katrina exemplifies what Klein (2007) calls “disaster capitalism”: the extreme profiteering from catastrophic events. The notion of “second-order disasters” is apt as it captures how recovery can cause more adverse effects than the original calamity (Adams, 2013). What has turned COVID-19 into a disaster is its spread in countries with depleted resources, inadequate public health policies, and underlying inequalities. It is still early days, but the vastly different outcomes among countries (in terms of deaths, but also secondary effects) suggest that social, political, economic, environmental, and cultural factors determine the course of the pandemic, not just the presence of the pathogen itself. In the essay, I will focus my discussion largely on the United Kingdom, which is one of the most severely affected countries globally.^1^

Digital technology has been at the center of the COVID-19 pandemic both globally and in the United Kingdom in particular. Not only have the lives of millions of people migrated online at a stroke as part of enforced lockdowns, digital innovation has also been integral to the public health response reflecting a well-established pattern which assumes that digital technologies and big data mitigate the harms caused by disasters. The empirical evidence, however, suggests the consequences of technology in disaster recovery are more complex at best and even harmful in some contexts. My earlier research of the aftermath of Typhoon Haiyan in the Philippines found that social and mobile media amplified already existing social inequalities leading to second-order disasters (Madianou, 2015).^2^ Popular assumptions that big data will provide “a single source of truth” that can guide government decisions (Gould et al., 2020) are cast into doubt when taking into account the epistemological and ontological limitations of crisis data (Crawford & Finn, 2015) such as the inherent biases and incompleteness of large data sets about disasters. What we observe during emergencies is a tendency to experiment with digital innovation without the usual public scrutiny (Madianou, 2019; Roberts, 2019) leading to concerns regarding privacy safeguards as happened during the Ebola epidemic (McDonald, 2016). The uses of machine learning and automation have increased the risks of discrimination in humanitarian emergencies (Madianou, 2019). I here argue that digital practices in the United Kingdom’s response to COVID-19 amplify social inequalities and can ultimately lead to second-order disasters.

The public health response to the COVID-19 pandemic led to the unprecedented situation of millions of people relying almost exclusively on communication technology to work, study, and socialize. It is probably fair to observe that there has never been such a heightened dependency on technology for such a wide range of activities at such a global scale. In the context of enforced physical distancing, digital media were a lifeline allowing elderly grandparents to interact with their grandchildren, friends to celebrate birthdays, family members to say goodbye to a relative quarantined in hospital. There are untold stories of care, love, and loss—as there are many stories when technology is a burden and source of stress, for example, when it erodes the boundaries between work and family life. What is clear is that any opportunities afforded by communication technologies are asymmetrically distributed. This is true for all spheres of social life, but is particularly relevant for education and work. In the United Kingdom, over a third (34%) of parents with children aged 5 to 16 reported that their child does not have access to their own computer or tablet at home (Montacute, 2020) which is vital to participate in distance learning. According to two recent studies, one fifth of UK pupils—over two million children—did no schoolwork at home, or less than an hour per day (Green, 2020), with children from better-off families spending 30% more time on home learning than are those from poorer families (Andrew et al., 2020). While virtually all (97%) privately schooled children had access to a computer at home, one in five of those on free school meals—a common indicator used to measure disadvantage in the United Kingdom—had no access. Children from the poorest families have been most affected by school closures, thus amplifying existing social inequalities.

Remote working also reveals stark asymmetries between those who can work from home and those whose jobs cannot be done remotely. It is no surprise that in the United Kingdom the highest rate of mortality is among working-class men and people from black and ethnic minority backgrounds who are most likely to be exposed to the virus due to the nature of their occupations, their dependency on public transport, and higher likelihood of underlying health conditions (Office for National Statistics, 2020) reflecting widespread health inequalities (Marmot, 2015). Data from the United States paint a similar picture (Taylor, 2020). By facilitating remote working only for a section of population, thus shielding them from the virus and allowing them to maintain their professional lives and income, digital technologies become part of a larger assemblage that perpetuates and increases social inequalities. This is one of the ways in which digital technologies become implicated with the stratified effects of the coronavirus.

Digital innovation and big data are also part of the public health response to the coronavirus pandemic. From contact tracing and symptom tracking apps to digital immunity certificates and quarantine enforcement surveillance systems, digital technologies are being deployed in the management of COVID-19. In dozens of countries, contact tracing apps—essentially tracking software installed on mobile devices that can determine contact between the user and any infected patients—have been rolled out as part of lockdown exit strategies.^3^ In the United Kingdom, digital contact tracing is developed by the innovation agency of the National Health Service (NHSX) and is expected to be rolled out in June 2020 as part of the government’s crisis exit strategy. The aim is for digital contact tracing to isolate clusters and avoid any virus flare-ups that will lead to blanket lockdowns. NHSX is also exploring digital immunity passports with private partners. According to some proposals, these would be a form of biometrically verified digital identity that confirms whether the user has COVID-19 antibodies.^4^ Significant concerns have been raised regarding privacy safeguarding, surveillance practices, and “function creep”: the reuse of data for different purposes than the ones for which they were originally collected. Furthermore, once surveillance infrastructures are established, it is difficult to dismantle them. The normalization of securitization post 9/11 is a case in point.

At the same time, serious reservations have been expressed about the effectiveness of these interventions. For example, the World Health Organization has cast serious doubt about immunity passports, mainly due to the lack of any conclusive scientific evidence regarding antibody-mediated immunity to SARS-CoV-2.^5^ Contact tracing apps, which have received the most widespread coverage in the United Kingdom and United States, are not deemed effective unless 56% of the population uses them (Hinch et al., 2020). Singapore, the first country to launch a contact tracing app in March 2020, saw a spike of cases after the app was rolled out. Only a fifth of the city-state’s population had downloaded the app^6^ while COVID-19 spread undetected in the cramped dormitories where migrant workers live—confirming that technological solutions cannot fix social inequalities.^7^ The list of the potential limitations of digital contact tracing is too long to detail here.^8^ What matters is that the concern over privacy and surveillance is valid whether contact tracing apps succeed in suppressing virus outbreaks or not. Even if digital contact tracing fails, the dissemination of such apps can still expand the power of the state and private companies as well as contribute to the entrenchment of surveillance. Reports have already highlighted how various contact tracing apps share data with private companies and governments. For example, the *Alerta Guarte* app in Guatemala shares sensitive user data with its US-based app developer as well as the national government which has explicitly stated that citizens should keep the app installed for further purposes such as security.^9^ The appropriation of the term “contact tracing” by the Minnesota Public Safety Commissioner to refer to the identification of potential suspects during the Black Lives Matter protests in the wake of George Floyd’s killing in May 2020 confirmed fears that “contact tracing” normalizes surveillance in spheres extending beyond the public health emergency.^10^ Inequality goes hand in hand with discrimination and surveillance as the latter has been systematically used on marginalized and minority people (Benjamin, 2019; Browne, 2015).

The digital response to the pandemic has been largely analyzed as a dramatic extension of state power.^11^ While states are responsible for public health policies, a closer look reveals the extensive involvement of the private sector. Digital innovation is almost always the result of public–private partnerships. In the United Kingdom, NHSX has partnerships with Amazon, Google, Microsoft, and Palantir Technologies.^12^ In addition to the state response, private firms such as PwC are rolling out their bespoke contact tracing app which they plan to make mandatory for employees returning to work.^13^ Such developments open the door for the monitoring of employees including after COVID-19. While much of the public debate on digital contact tracing has focused on privacy and surveillance concerns, one the most compelling consequences of this innovation is the way it entrenches the “digital welfare state,” a term that is used to refer how the systems of “social protection and assistance are increasingly driven by digital data and technologies that are used to automate, predict, identify, surveil, detect, target and punish” (Alston, 2019). While this definition captures the digitization of welfare, it does not highlight the fact the digital welfare state is also increasingly privatized. Large technology companies are responsible for providing the hardware or computational systems that underpin automation, identification, and surveillance.

We discern a number of different logics by dissecting public–private partnerships: big technology companies are driven by a logic of profit and desire for growth. Conversely, the state is driven by the imperative to manage the disease as well as a logic of control. Also present is the logic of solutionism: the desire to find technological solutions to complex social problems. Solutionism is very attractive especially in the absence of a clear exit from the pandemic, such as a vaccine or an effective drug treatment. Given the impending economic downturn predicted to ensue from the crisis, technological solutions provide governments with a tangible thing that they seem to be doing. At the same time, the logic of profit undermines public institutions. By providing services or licensing products to run public services, technology companies hollow out the infrastructures of the welfare state, ultimately leaving them weakened. This structural transformation, the consolidation of the digital and privatized welfare state, is one of the most critical dimensions of the digital response to the COVID-19 crisis.

Technology companies have used the pandemic as an opportunity to extend their reach well beyond the public health response. The massive experiment of millions of people migrating online by default has been seized by companies which see opportunities not only for profit, but also for entrenching themselves in public life. The most compelling evidence of this new wave of “disaster capitalism” came when the Governor of New York State, Andrew Cuomo, appointed Eric Schmidt, the former CEO of Google, to head a panel that will radically rethink the New York City after COVID-19. This “screen new deal,” according to Naomi Klein (2020), involves the promotion of online education, telemedicine, and smart city infrastructure among other things which will increase opportunities for data extraction and profit (Zuboff, 2019). At the same time, this signals a further privatization of public space and public institutions (schools, universities, hospitals). The irony is that just weeks before the pandemic took hold, technology companies were heavily criticized for a litany of failings including their lack of accountability. The “shock doctrine” (Klein, 2007) that follows all disasters stifled criticisms and quickly embraced technology as a “solution” to life in lockdown and beyond.

I argued some years ago that digital technology contributes to “second-order disasters”—the human-made disasters that trap already disadvantaged people into precarity (Madianou, 2015). So far, the digital response to COVID-19 appears to be no exception. The evidence from the United Kingdom and around the world shows that the patterns from previous disasters are being repeated. The uses of technology amplify existing inequalities, which are already accentuated by the pandemic. This is apparent in the spheres of education and work where digital technology is deeply implicated with the stratified effects of lockdown policies. The use of technologies in the public health response further reproduces inequalities. Experimentation with technology carries privacy risks and raises concerns about data sharing with states and private companies. Applications such as contact tracing apps normalize surveillance which has been traditionally used on marginalized communities. The outsourcing of the digital public health response consolidates the arrival of the digital welfare state, which is increasingly privatized. The combined digitization and privatization of welfare signals a hollowing out of public institutions—not just welfare but also schools, universities, cities which will in turn further accentuate inequalities and potential discrimination through automation and algorithmic filtering (Benjamin, 2019). The article has focused mainly in the United Kingdom, but some of the examples examined suggest that the argument may be applicable to other contexts.

There is a further secondary effect: placing so much emphasis on technological solutions risks depoliticizing the COVID-19 emergency. The logic of technological solutionism has the capacity to occlude the workings of technology and digital capitalism with extraordinary ease (Madianou, 2019). This matters because now, more than ever, there is an imperative to collectively reimagine the future after the pandemic. And this is a deeply political task.

## Notes

1. At the time of writing, the United Kingdom had the highest death toll on Europe and the second highest in the world after the United States. For a list of all COVID-19 cases globally, see https://gisanddata.maps.arcgis.com/apps/opsdashboard/index.html#/bda7594740fd40299423467b48e9ecf6

2. That research found that during the delayed recovery the vast majority of low-income participants were pushed further into poverty. For example, one year after the Typhoon made landfall, the majority of research participants were unemployed and continued to live in temporary accommodation. Their social media uses, while creative, did not offer any significant opportunities for a redistribution of resources. Only a very small number of middle-class professionals were able to exploit opportunities in social media to improve their economic and social standing (Madianou, 2015).

3. For a list of all digital contact tracing projects to date, see https://www.technologyreview.com/2020/05/07/1000961/launching-mittr-covid-tracing-tracker/

4.
https://www.technologyreview.com/2020/05/07/1000961/launching-mittr-covid-tracing-tracker/

5.
https://www.who.int/news-room/commentaries/detail/immunity-passports-in-the-context-of-covid-19

6.
https://qz.com/1842200/singapore-wants-everyone-to-download-covid-19-contact-tracing-apps/

7. A new contact tracing app was made mandatory for migrant workers in Singapore at the end of May 2020, raising additional concerns regarding consent, the right to “opt out” and potential stigmatization (as the app is only mandatory for migrants) https://privacyinternational.org/examples/3890/singapore-contact-tracing-app-made-mandatory-migrant-workers

8. For a review see the report by the Ada Lovelace Institute (2020).

9. https://privacyinternational.org/examples/3660/guatemalan-health-information-app-collects-personal-data. See the work of Privacy International more broadly for reports on the digital response to covid-19.

10.
https://www.vox.com/recode/2020/6/1/21277393/minnesota-protesters-contact-tracing-covid-19

11. See *The Economist*, “Everything is under control: the state in covid-19”: https://www.economist.com/leaders/2020/03/26/the-state-in-the-time-of-covid-19

12.
https://healthtech.blog.gov.uk/2020/03/28/the-power-of-data-in-a-pandemic/

13.
https://www.ft.com/content/caeb250b-8d8b-4eaa-969c-62a8b58464aa
